# The Impact of Elevated Printing Speeds and Filament Color on the Dimensional Precision and Tensile Properties of FDM-Printed PLA Specimens

**DOI:** 10.3390/polym17152090

**Published:** 2025-07-30

**Authors:** Deian Dorel Ardeljan, Doina Frunzaverde, Vasile Cojocaru, Raul Rusalin Turiac, Nicoleta Bacescu, Costel Relu Ciubotariu, Gabriela Marginean

**Affiliations:** 1 Department of Engineering Science, Babeș-Bolyai University, Traian Vuia Square 1–4, 320085 Reșița, Romania; deian.ardeljan@stud.ubbcluj.ro (D.D.A.); raul.turiac@ubbcluj.ro (R.R.T.); nicoleta.bacescu@ubbcluj.ro (N.B.); relu.ciubotariu@ubbcluj.ro (C.R.C.); 2 Institute of Mechanical Engineering, Westphalian University of Applied Sciences Gelsenkirchen Bocholt Recklinghausen, Neidenburger Str. 43, 45897 Gelsenkirchen, Germany; gabriela.marginean@w-hs.de

**Keywords:** additive manufacturing (AM), polylactic acid (PLA), fused filament fabrication (FFF), fused deposition modeling (FDM), printing speed, filament color, dimensional accuracy, ultimate tensile strength (UTS)

## Abstract

This study examines the effect of elevated printing speeds (100–600 mm/s) on the dimensional accuracy and tensile strength of PLA components fabricated via fused deposition modeling (FDM). To isolate the influence of printing speed, all other parameters were kept constant, and two filament variants—natural (unpigmented) and black PLA—were analyzed. ISO 527-2 type 1A specimens were produced and tested for dimensional deviations and ultimate tensile strength (UTS). The results indicate that printing speed has a marked impact on both geometric precision and mechanical performance. The optimal speed of 300 mm/s provided the best compromise between dimensional accuracy and tensile strength for both filaments. At speeds below 300 mm/s, under-extrusion caused weak layer bonding and air gaps, while speeds above 300 mm/s led to over-extrusion and structural defects due to thermal stress and rapid cooling. Black PLA yielded better dimensional accuracy at higher speeds, with cross-sectional deviations between 2.76% and 5.33%, while natural PLA showed larger deviations of up to 8.63%. However, natural PLA exhibited superior tensile strength, reaching up to 46.59 MPa, with black PLA showing up to 13.16% lower UTS values. The findings emphasize the importance of speed tuning and material selection for achieving high-quality, reliable, and efficient FDM prints.

## 1. Introduction

Over the past few decades, additive manufacturing (AM), widely referred to as 3D printing, has become a transformative technology across numerous industries. In contrast to conventional subtractive manufacturing—which removes material from a solid block—AM constructs objects layer by layer based on digital models. This approach offers exceptional design flexibility, improved material efficiency, and a high degree of customization. Originally developed for rapid prototyping, AM has evolved into a robust production technology with applications in aerospace, healthcare, automotive, construction, and consumer goods [1,2].

The growing importance of additive manufacturing is driven by several converging factors: advancements in materials science, improvements in printing technologies, and the increasing demand for sustainable and decentralized production systems. As global supply chains face mounting pressures from geopolitical tensions, environmental concerns, and shifting consumer expectations, AM offers a compelling alternative that supports localized, on-demand manufacturing with reduced waste and lead times [3]. Furthermore, the integration of AM into Industry 4.0 frameworks and circular economy models underscores its strategic value in enabling more resilient and environmentally conscious production paradigms [1].

Additive manufacturing encompasses a variety of technologies, each with distinct mechanisms, materials, and applications [4]. Among the AM processes, fused deposition modeling (FDM) has distinguished itself due to its cost-effectiveness, user-friendly nature, and versatility in processing various thermoplastic materials. The process functions by extruding heated filament through a controlled nozzle, depositing material sequentially in layers to construct the final three-dimensional structure.

Despite its popularity, FDM faces limitations in terms of surface finish, mechanical strength, and dimensional accuracy. However, ongoing research continues to improve FDM through material innovations and process optimization, making it increasingly viable for functional parts and industrial applications. Recent studies have demonstrated that optimizing FDM parameters such as layer height and infill density can significantly enhance mechanical performance, even when using recycled materials like rPETG, thus supporting both engineering functionality and sustainability goals [5]. Its accessibility and adaptability have positioned FDM as a cornerstone of the AM ecosystem, particularly in contexts where cost efficiency and rapid iteration are critical.

Among the filament types utilized in FDM, Polylactic Acid (PLA) is notable for its biodegradability, ease of processing, and compatibility with low-cost printers. Recent studies have shown that thermal post-processing can significantly enhance PLA’s mechanical performance, making it more suitable for functional applications while maintaining its environmental advantages [6].

A critical aspect of FDM’s performance lies in the large number of interdependent process parameters that influence the surface quality [7,8], strength [9,10,11,12,13], and dimensional accuracy [14,15,16,17] of printed parts. Parameters such as nozzle temperature, temperature of the build plate, layer height, printing speed, infill density, build orientation, and cooling rate must be finely adjusted to exhibit the targeted properties, while simultaneously reducing build time and minimizing material consumption, as shown by Maalihan et al. [18]. The sensitivity of FDM outputs to these variables makes the process both highly customizable and technically demanding. As noted by Ponis et al. [1], the complexity of AM processes like FDM requires a deep understanding of parameter interactions to ensure consistent outcomes. On the other hand, varying multiple process parameters simultaneously during the experiments makes it challenging to isolate and identify the individual effect of each parameter, as concluded by Cojocaru et al. [9]. As AM transitions from prototyping to end-use part production, the optimization of these parameters has become a key research focus.

Rapid manufacturing comes not only with demands regarding predictability of properties, surface quality, and dimensional accuracy, but also productivity and cost-effectiveness. In order to shorten the production times, two solutions could be adopted: simultaneous printing of identical parts or printing with high printing speeds. As for the first, there are space limitations regarding the number of prints that might be produced at the same time on the build platform. Moreover, one has to take into account the alteration of both the dimensional accuracy and the mechanical strength of parts printed in batches relative to individually printed parts, and the non-uniformity of the properties between the prints belonging to the same batch, as revealed by previous research [19].

Considering the above, enhancing the printing speed appears to be the better solution for increasing productivity and reducing energy consumption. In fused deposition modeling (FDM), printing speed refers to the linear velocity at which the printer’s nozzle moves while extruding thermoplastic material to form each layer of a 3D printed object. It has to be distinguished from travel speed, which refers to the nozzle’s movement when not extruding material.

The printing speed is typically measured in millimeters per second (mm/s) and directly influences the build time, dimensional accuracy [20,21], surface quality [21], and mechanical properties of the final part [9,22]. Optimal printing speed settings depend on factors such as material type, nozzle diameter, layer height, printing head temperature, and cooling efficiency, and are typically in the range of 20–170 mm/s for standard PLA prints [9].

To ensure consistent material deposition and part quality, the printing speed is a process parameter that must be carefully controlled. While higher printing speeds shorten production time, they often lead to reduced interlayer adhesion, increased surface roughness, greater dimensional deviations, and weaker mechanical properties because of increased vibrations and less precise control over the extrusion process [20,23,24]. Conversely, lower speeds allow for more controlled material deposition and better fusion between layers due to longer cooling and bonding times, enhancing both accuracy and strength—but at the cost of longer print durations. Previous research indicates that moderate printing speeds, typically between 40 and 60 mm/s, offer the best compromise [17,20,22,24,25,26]. At these speeds, PLA parts exhibit improved dimensional accuracy and surface quality while maintaining acceptable mechanical strength.

As the general trend suggests that higher speeds tend to compromise dimensions, surface quality, and mechanical properties, most studies focus on optimizing within lower speed ranges for better strength and efficiency. While there is substantial research on optimizing FDM printing parameters for PLA, including print speeds up to around 170 mm/s [9], only one previous study could be found that investigates specific higher printing speeds [27]. In their work, Lorkowski et al. [27] compared the effects of two printing speeds (30 mm/s and 500 mm/s, respectively) on the mechanical strength and surface quality of PLA samples, also considering the influence of the orientation of the samples on the build plate and post-processing by ironing. They concluded that higher printing speeds can significantly reduce both production time and energy consumption, with only a minimal influence on the mechanical performances of the prints—assuming filaments specifically designed for high-speed printing are used.

Among the FDM process parameters, a less targeted parameter is the cooling speed. Its role has to be carefully considered in case of higher printing speeds.

The cooling mechanism directly affects the thermal history of the printed layers, which in turn governs the dimensional accuracy and mechanical performance of the final product. Studies have shown that higher cooling speeds, typically achieved through forced-air systems, enhance the geometric precision of PLA prints by minimizing thermal deformation and warping [28]. This improvement in dimensional accuracy is attributed to the rapid solidification of extruded material, which stabilizes the printed geometry more effectively [28].

Conversely, the mechanical integrity of PLA components, particularly tensile strength, tends to diminish with increased cooling rates. Rapid cooling can hinder adequate interlayer bonding, resulting in weaker structural cohesion [28]. Experimental findings indicate that PLA specimens subjected to high cooling velocities exhibit tensile strengths significantly lower than those cooled at ambient conditions [28]. Furthermore, the cooling rate influences the crystallinity of PLA, with slower cooling promoting higher crystalline content and, consequently, improved mechanical properties [29].

To achieve an optimal balance between dimensional accuracy and tensile strength, it is essential to fine-tune the cooling parameters. Moderate cooling speeds may offer a compromise that ensures sufficient geometric fidelity without severely compromising mechanical robustness [28]. Additionally, other process variables such as layer thickness, print speed, and extrusion temperature interact with cooling dynamics and must be considered in a holistic optimization strategy [22,30,31].

While numerous studies have explored the impact of process parameters such as layer height, infill density, and printing temperature on the dimensional accuracy and mechanical strength of FDM-printed parts, the influence of filament color remains comparatively underexplored and often unreported in experimental setups [14,16,17]. Recent studies, however, have shown that the color of PLA filaments can have a significant impact on both the dimensional accuracy and mechanical properties of printed parts, even when all other printing parameters remain unchanged [14,16,17,19,32].

These differences are attributed to the varying additives and pigments added to produce various filament colors, which can influence the material’s thermal and physical behavior during the printing process [14,16,31]. Experimental investigations have demonstrated that PLA color can lead to measurable variations in dimensional accuracy, with certain colors, such as black, providing superior precision, while others, like gray, may yield higher tensile strength values [16,17,19]. Furthermore, the mechanical characteristics, including tensile, compressive, and flexural strengths, have been shown to fluctuate depending on the filament color, with some studies reporting up to a 10% difference in tensile strength between colors under identical printing scenarios [19,32,33].

The underlying causes for these differences are believed to be related to the color-dependent crystallinity that develops during processing and/or variations in thermal behavior [34,35] observed in PLA filaments with added colorants. Some researchers have provided more detailed explanations, suggesting that these coloring agents may influence the crystallization rate [36], act as nucleating agents [37], or hinder material flow during the printing process [38].

Given the growing demand for reliable and high-performance 3D printed parts in engineering and consumer applications, understanding the role of filament color is essential for optimizing print quality and ensuring the desired mechanical performance.

Printing time is a critical parameter in additive manufacturing, both in the context of rapid prototyping and rapid fabrication. Minimizing print duration accelerates design iterations, reduces development cycles, reduces energy consumption, and enhances production efficiency. On the other hand, increasing print speed often involves trade-offs in surface quality, dimensional accuracy, and mechanical performance. Considering all the findings mentioned above, but also the lack of information regarding printing speeds above 170 mm/s, this study aims to systematically investigate the influence of high printing speeds, situated between 100 and 600 mm/s, on the dimensional accuracy of PLA parts produced via FDM, while also considering its implications for mechanical performance. Understanding this relationship is essential for optimizing print settings to achieve high-quality, reliable, and time-efficient 3D printed components. To isolate the material response to increased printing speeds, all other printing parameters were kept constant, as detailed below.

As the effect of high printing speeds on the print quality strongly depends on the thermal properties of the PLA filament, the experiments were conducted on two PLA colors: natural (plain) PLA, without any coloring additives, and black PLA. The latter was chosen as it was expected that the PLA filament obtained by the addition of carbon black might manifest a significantly accelerated response to the rapid variations in the thermal printing conditions, as it was demonstrated by previous studies that the black filler material (carbon black) imparts its conductivity to PLA [34,39].

## 2. Materials and Methods

The investigation into the effects of printing speed and filament color on the dimensional accuracy and mechanical strength of components fabricated via fused deposition modeling (FDM) was conducted using ISO 527-2 type 1A [40] specimens (4 mm nominal thickness).

The specimen’s CAD model was designed using SolidWorks 2024 (Dassault Systèmes, Vélizy-Villacoublay, France), and the slicing parameters were generated with the Orca Slicer v2.3.0 (SoftFever, Open-source project, GitHub platform [41]). Printing was carried out on an FLSUN T1 Pro delta FDM printer (FLSUN, Zhengzhou, China) that features a fully enclosed build chamber (Ø260 × 330 mm). Because PLA material was used for the experiments, the enclosure door was deliberately left open during testing to prevent excessive heat build-up, while still taking advantage of the printer’s bilateral continuous positive airway pressure fan cooling system (CPAP fan). The machine is equipped with a 0.4 mm brass nozzle. With this nozzle, the attainable layer height can be adjusted from 0.35 mm down to 0.10 mm [38]. Part cooling is provided by the factory-installed Silent Module, which integrates a high-flow CPAP turbo fan (~30 000 rpm) and enlarged bilateral air ducts, reducing noise to ~55 dB, while ensuring uniform layer solidification [42,43].

Table 1 shows the process parameters used for the printing of the samples. They were set following the recommendations of the filament supplier. The only variables were the printing speed—set at 100 mm/s, 200 mm/s, 300 mm/s, 400 mm/s, 500 mm/s, and 600 mm/s—and the filament color, with two variants: natural and black. The PLA filaments utilized were “Filament 1.75 mm EasyFil ePLA Natural” and “Filament 1.75 mm EasyFil ePLA Traffic Black”, both produced by FormFutura 3D Printing Materials (FormFutura B.V., Nijmegen, The Netherlands). According to the manufacturer’s specifications, these materials exhibit a tensile strength at break of 53 MPa, a tensile modulus of 3.5 MPa, a heat deflection temperature of 55 °C, and a glass transition temperature of 60 °C [44]. While compatible with standard FDM 3D printers, these filaments are specifically optimized for high-speed 3D printing technologies. For each filament color, 42 specimens were fabricated—7 for each printing speed—leading to a total of 84 specimens. All specimens were printed individually—one per build plate—in YX orientation, as defined by ISO/ASTM 52921:2013 [45]. No pre-processing of the filament or post-processing of the printed specimens was performed. Prior to printing, the PLA filament spools were stored in sealed packaging to prevent moisture absorption and exposure to ultraviolet radiation.

**Figure 1:**
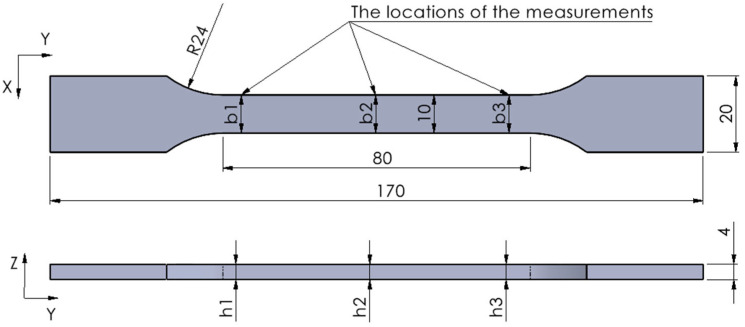
The CAD model of the tensile test specimens: b_1_, b_2_, b_3_—positions for width measurements; h_1_, h_2_, h_3_—positions for thickness measurements.

Width and thickness of the tensile specimens were measured at three locations along the calibrated section to assess dimensional accuracy, as shown in Figure 1.

Measurements were performed using a Mitutoyo 293-240-30 digital micrometer (Mitutoyo România SRL, Otopeni, Romania), featuring a measuring range of 0–25 mm and an accuracy of ±0.001 mm. For each specimen, dimensional deviations (expressed as percentages) were calculated for the width measurements (b_1_, b_2_, b_3_) and thickness measurements (h_1_, h_2_, h_3_). The average deviations in both width and thickness were subsequently calculated and graphically illustrated. Furthermore, the mean deviations in cross-sectional area, relative to the nominal value of 40 mm^2^, were evaluated for each specimen group.

Tensile testing was conducted using a Mecmesin Multitest 2.5dV universal testing machine (PPT Group UK Ltd., Slinfold, UK), equipped with a 2500 N load cell and operated via the Vector ProMT 6.1.0.0 control software. The tests were performed in accordance with ISO 527-1 [46] and ISO 527-2 [40], employing a constant crosshead speed of 10 mm/min. For each group of specimens, the ultimate tensile strength (UTS) and corresponding standard deviations were calculated.

The mesostructural characteristics of the specimens were examined on both the fracture surfaces and the top-layer surfaces using a Leica MZ 7.5 stereomicroscope (Leica Microsystems, Wetzlar, Germany) at a magnification of 10×.

## 3. Results and Discussions

The experimental results, highlighting the influence of printing speed and filament color on the dimensional accuracy and tensile strength of PLA specimens produced using fused deposition modeling (FDM), are presented and analyzed in the following subsections. To facilitate the visual identification of color-dependent trends, all graphical representations employ a consistent color scheme: yellow for natural PLA and dark gray for black PLA. These colors are used to denote the dimensional deviations and ultimate tensile strength (UTS) values corresponding to each filament type.

### 3.1. Dimensional Accuracy

**Figure 2:**
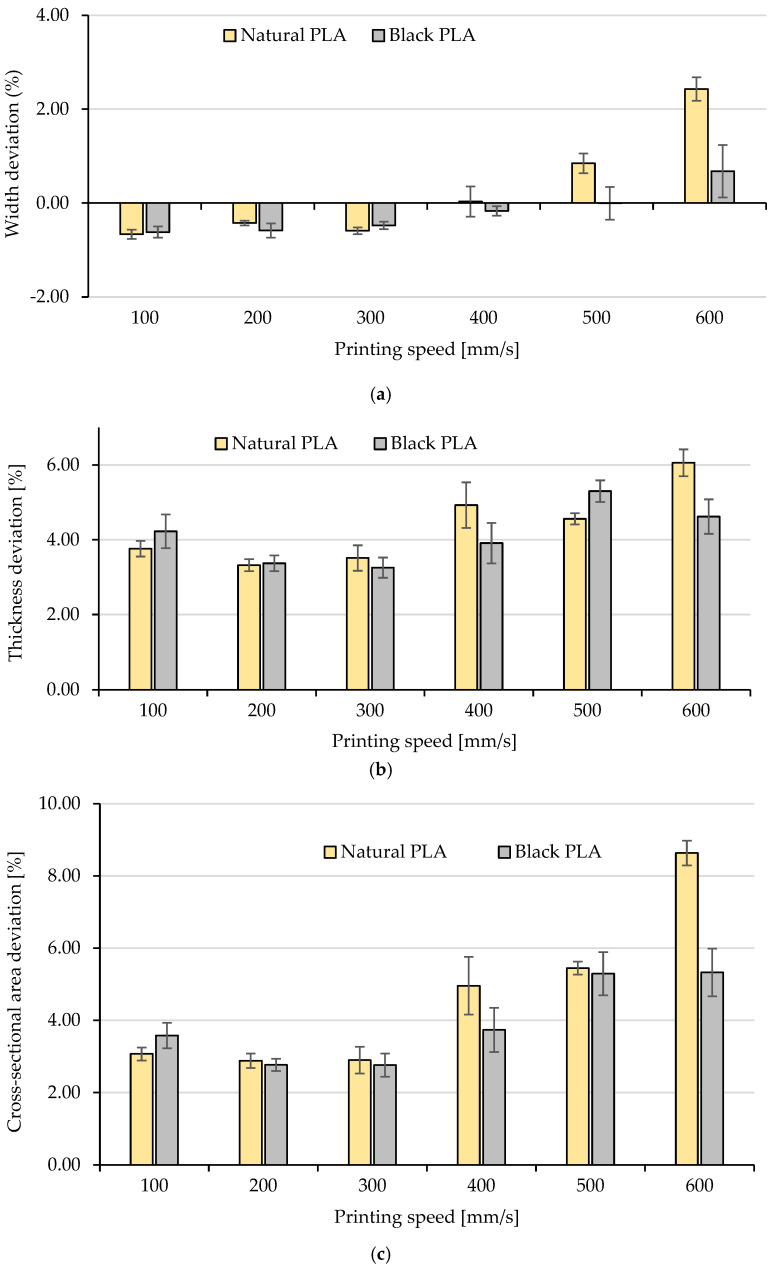
Dimensional deviations of test specimens manufactured at varying printing speeds: (**a**) with deviations (nominal value = 10 mm); (**b**) thickness deviations (reference value = 3 mm); (**c**) deviations in the cross-sectional area (reference value = 40 mm^2^).

To highlight the influence of printing speed on dimensional accuracy and the potential variability introduced by filler materials in colored PLA filaments, dimensional deviations in width, thickness, and cross-sectional area within the calibrated region of the test specimens were quantified and are presented in Figure 2.

As one can observe in Figure 2a,b, the dimensional deviations of the specimens’ width (Figure 2a), which ranged from −0.66% (s_p_ = 100 mm/s) to 2.43% (s_p_ = 600 mm/s) for natural PLA and from −0.62% (s_p_ = 100 mm/s) to 0.68% (s_p_ = 600 mm/s) for black PLA, were considerably smaller than those observed in the thickness (Figure 2b), where deviations ranged between 3.32% (s_p_ = 200 mm/s) and 6.06% (s_p_ = 600 mm/s) for natural PLA and between 3.25% (s_p_ = 300 mm/s) and 5.30% (s_p_ = 500 mm/s) for black PLA. These results are consistent with the findings reported in [17], as this discrepancy can be attributed to the fact that the specimens were able to expand more freely in the vertical direction (*Z*-axis, corresponding to thickness), whereas lateral expansion (*X*-axis, corresponding to width) was constrained by the presence of the two wall lines forming the outer shell of the printed parts.

In previous research, when using standard or optimized process parameters and moderate printing speeds, the dimensional deviations for FDM-printed PLA parts were generally found to be below 5% for most features and shapes [47,48]. Moreover, for different colored dog-bone tensile test specimens, printed at 50 mm/s [16] and 60 mm/s [44], with different layer heights [16] and raster angles [49], width deviations ranged between 0.17 and 0.75%, respectively, significantly lower than height or thickness deviations (up to 5–9.5%) [16,49]. Consequently, one may conclude that both the width and thickness deviations obtained by printing with high speeds (100–600 mm/s) are not falling outside the usual value ranges for FDM printing of PLA with moderate printing speeds, although the printing time was shortened from 26 min 26 s (100 mm/s) to 5 min 6 s (600 mm/s). The sole exception was recorded in the width deviations of natural PLA printed at 600 mm/s, which reached 2.43%; however, this value remains below the general threshold of 5%.

Comparing the effect of the filament color on the samples’ dimensional accuracy across varying printing speeds, the experimental data shown in Figure 2c (overall accuracy visualized by the cross-section deviations) indicate that, also at high speeds, different colored PLA filaments respond differently to identical printing conditions. So, except for the lowest printing speed tested (100 mm/s), black PLA exhibited better overall dimensional accuracy than natural PLA. The deviations in cross-sectional dimensions for natural PLA prints ranged from 2.88% at 200 mm/s to 8.63% at 600 mm/s, while the variation interval for black PLA was between 2.76% (200 mm/s) and 5.33% (600 mm/s). These variations may be attributed to the distinct thermal and physical properties of the different colored filaments, since prior research has shown that the glass transition temperature [35] and the conductivity of PLA [34] may differ as a consequence of the coloring agents.

Although the magnitude of the dimensional deviations is clearly influenced by the filament color, the trend of variation with increasing printing speed—from 100 mm/s to 600 mm/s—was analogous for both series of samples (natural PLA, respectively, black PLA). At printing speeds between 100 and 300 mm/s, the overall dimensional accuracy (expressed by cross-sectional area deviations, Figure 2c) improved for all samples with increasing speed up to 300 mm/s and then worsened as the speed was raised to 600 mm/s. Both PLA filament types exhibited optimal overall dimensional accuracy and minimal values in the cross-sectional area deviations at printing speeds around 300 mm/s.

In terms of width accuracy, for both filament colors, the deviations along the X direction changed the sign from negative (−) to positive (+) values, for printing speeds between 300 mm/s and 400 mm/s for natural PLA and around 500 mm/s for black PLA (Figure 2a). On the other hand, the height deviations were positive for all printing speeds and both filament colors, but their dependence on the printing speed was not linear (Figure 2b), also determining the non-linear variations in the cross-sectional area deviations in relation to the printing speed. For natural PLA, the minimum value of the height deviations was recorded at 200 mm/s and, for black PLA, at 300 mm/s, while all other printing speeds resulted in poorer thickness accuracy.

**Figure 3:**
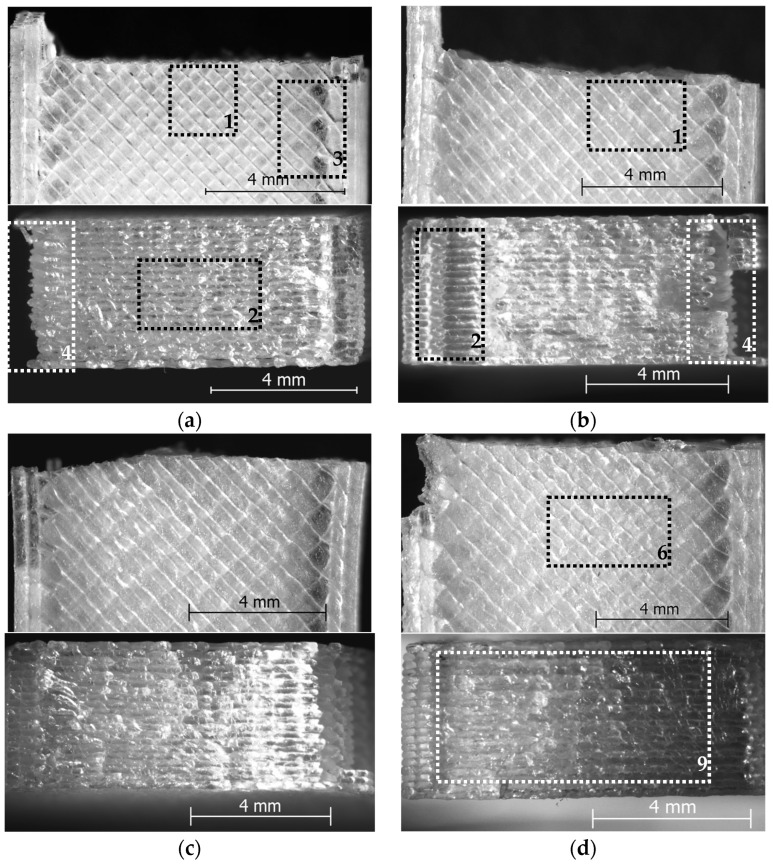

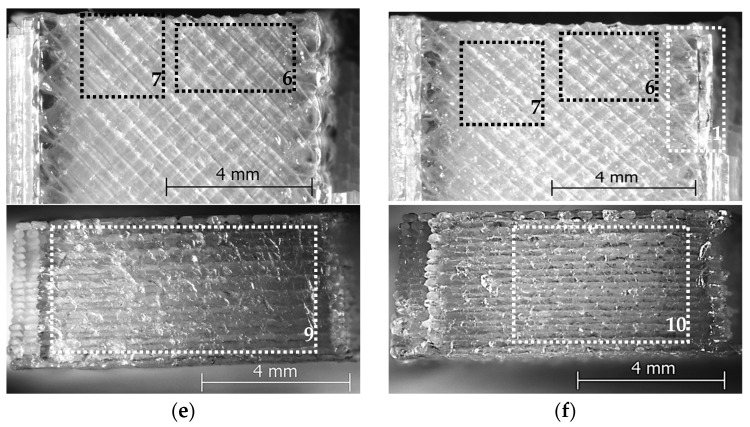
Top-view (above) and fractured surface (below) of natural PLA specimens fabricated at (**a**) v = 100 mm/s, (**b**) v = 200 mm/s, (**c**) v = 300 mm/s, (**d**) v = 400 mm/s, (**e**) v = 500 mm/s, (**f**) v = 600 mm/s.

**Figure 4:**
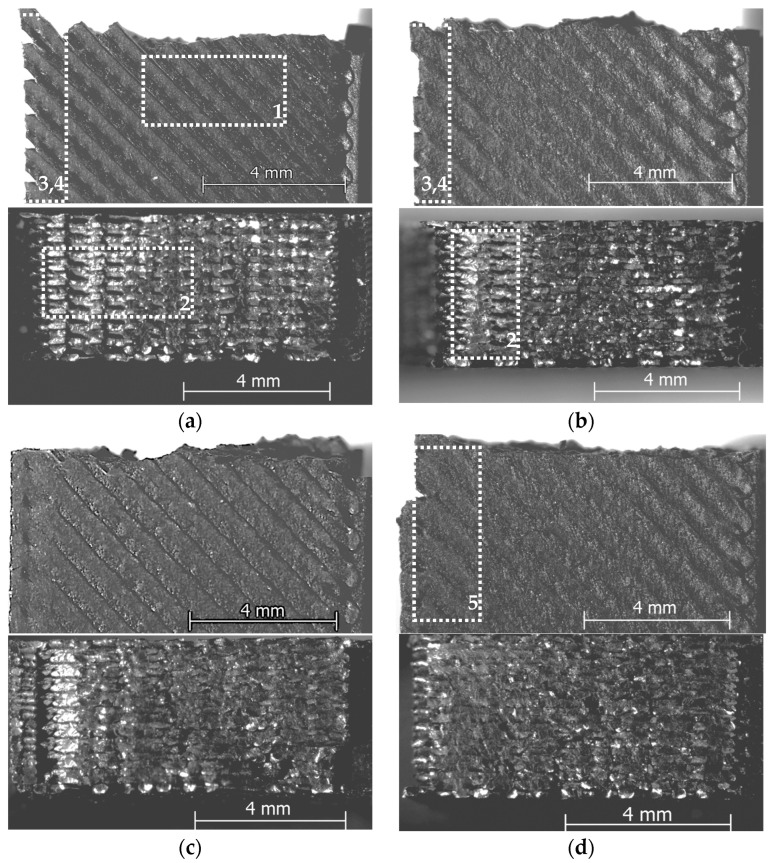

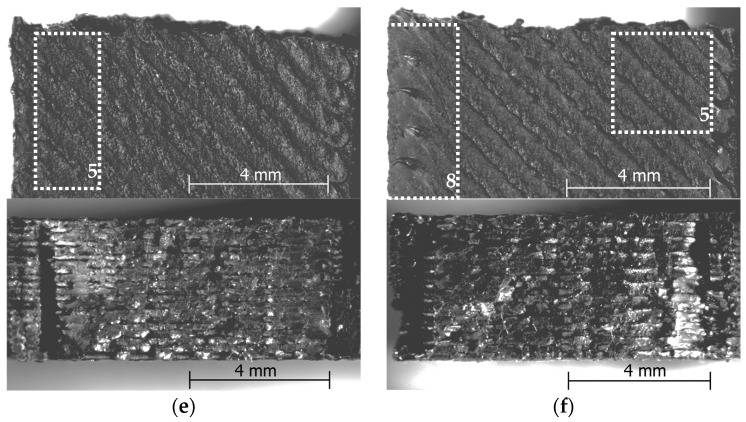
Top-view (above) and fractured surface (below) of black PLA specimens fabricated at (**a**) v = 100 mm/s, (**b**) v = 200 mm/s, (**c**) v = 300 mm/s, (**d**) v = 400 mm/s, (**e**) v = 500 mm/s, (**f**) v = 600 mm/s.

To explore the fundamental reasons behind these outcomes, both the surface morphology and the specimens’ fracture surfaces (after tensile testing) were analyzed through light microscopy, as outlined in Section 2. Representative micrographs for each color–printing speed combination are displayed in Figure 3 (natural PLA) and Figure 4 (black PLA), respectively.

As illustrated in Figure 3 and Figure 4, for both filament colors, increasing the printing speed within the range of 100–600 mm/s resulted in distinct surface and cross-sectional characteristics of the tensile specimens. These ranged from signs of under-extrusion—attributed to insufficient temperatures during the printing process (Figure 3a,b and Figure 4a,b)—to indications of over-extrusion, caused by excessively high temperatures (Figure 3d–f and Figure 4d–f). Only the 300 mm/s printing speed ensured acceptable surface and cross-section structures at the maximal speed of the CPAP fan (100%, Table 1).

So, in case of the printing speeds set at 100 mm/s and 200 mm/s, one can observe rough surfaces, showing narrow, pinched filament tracks (marked with 1 in Figure 3a,b and Figure 4a), air gaps between successive layers (marked with 2 in Figure 3a,b and Figure 4a,b), and respective contour lines and infill (marked with 3 in Figure 3a and Figure 4a,b). Moreover, during tensile testing, the contour lines detached from the infill (regions 4 in Figure 3a,b and Figure 4a,b). These effects were also shown in previous research [50,51,52,53,54] to be caused by improper thermal conditions that led to under-extrusion during FDM printing. Weak bonding between layers determines size increase along the *Z*-axis, where the material may expand freely. Conversely, under-extrusion typically leads to narrower surface tracks and thereby to smaller part dimensions in width, especially in the X and Y directions of FDM-printed PLA parts, as also shown by Jakupi et al. [54].

On the other hand, the printing speeds of 400 mm/s, 500 mm/s and 600 mm/s, as shown by Figure 3d–f (natural PLA) and Figure 4d–f (black PLA), respectively, reveal sample surfaces with filament flattening (marked with 5 in Figure 4d–f), non-uniform filament roads (marked with 6 in Figure 3d–f), bulges (marked with 7 in Figure 3d,e), loss of sharp details (marked with 8 in Figure 4f), and cross-sections with extremely dense mesostructures (marked with 9 in Figure 3d,f), suggesting that the temperature was consistently maintained at an excessively high level during the printing process, leading to thermal printing conditions considered to be specific for over-extrusion [50,51]. Interestingly, in case of the natural PLA printed at 600 mm/s, where the highest dimensional deviations were recorded, both in width and thickness, specific aspect of the samples surface indicates to high printing temperature, while the cross-section shows delamination (marked with 10 in Figure 3f) and critical contour detachment (marked with 11 in Figure 3f). Printing at very high speeds combined with intense bilateral cooling induces a rapid sequence of heating and cooling cycles during the material deposition process. This results in steep thermal gradients, which contribute to the accumulation of internal stresses and subsequent distortions within or between layers, ultimately leading to interlayer cracking, as shown by previous research [55,56]. These thermal and mechanical effects likely account for the increased dimensional deviations observed in both width and height, as also noted by the authors in [57].

The highest overall dimensional accuracy for both types of PLA filaments was achieved at a printing speed of 300 mm/s (Figure 2c). In accordance with these results, Figure 3c and Figure 4c reveal acceptable structures for the surfaces and cross-section of the tested samples, respectively.

Considering the above, the variation curves of the dimensional deviations (width, thickness, and cross-sectional area), with minimum values around the printing speed of 300 mm/s and increasing values in both directions, respectively, for lower (200 mm/s to 100 mm/s) and higher printing speeds (400 mm/s to 600 mm/s) are fully sustained by the structural aspect of the tensile samples surfaces and cross-sections.

### 3.2. Tensile Behavior

**Figure 5:**
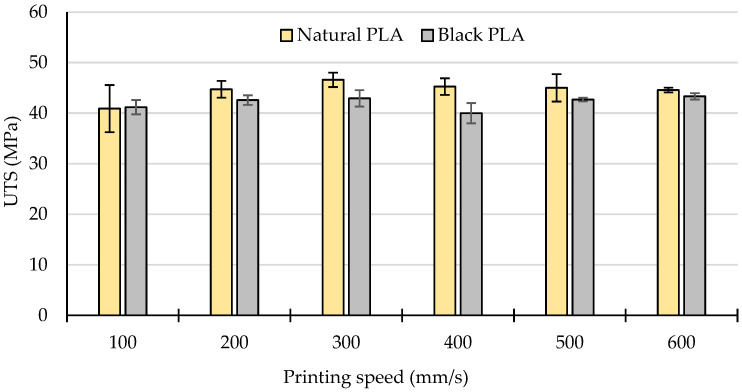
The variation in the ultimate tensile strength (UTS) with the printing speed and the filament color.

The average values of ultimate tensile strength (UTS), along with the corresponding standard deviations, for specimens fabricated at varying printing speeds and using different filament colors, are illustrated in Figure 5. As previously mentioned, each color–speed combination was represented by a set of seven tensile specimens. To minimize the influence of outliers and ensure consistency in the analysis, the minimum and maximum values within each set were excluded from the statistical evaluation.

As illustrated in Figure 5, the UTS values of both types of filaments, printed with speeds between 100 mm/s and 600 mm/s, ranged between 40.00 MPa (black PLA, s_p_ = 400 mm/s) and 46.59 MPa (natural PLA, s_p_ = 300 mm/s). As reported in previous studies [58,59,60], standard PLA prints produced under optimized conditions and usual printing speeds (30–125 mm/s) typically exhibit UTS values ranging from 24 to 47 MPa. Therefore, the UTS achieved at elevated printing speeds up to 600 mm/s can be considered to lie within the upper limit of this typical range.

The general trend of the ultimate tensile strength (UTS) as a function of the printing speed remained consistent for both filament colors: the UTS increased with printing speeds up to 300 mm/s, after which it declined as the speed continued to rise to 600 mm/s. The observed variation in UTS values corresponds with the mesostructural features of the fractured surfaces shown in Figure 3 (natural PLA) and Figure 4 (black PLA), showing a continuous increase in the mechanical strength for printing speeds as the bond between deposited filaments increased, followed by a recrudescence of the UTS in the domain of the printing speeds exceeding 300 mm/s, which was due to over-extrusion and layer delamination caused by internal stresses. This trend aligns with the observations of Lendvai et al. [61], who reported that increasing the filament extrusion multiplier (k) above 0.97 reduced void content and porosity—thereby enhancing tensile strength—while values exceeding 1.05 led to over-extrusion, introducing stress concentration sites that ultimately diminished the mechanical resistance.

On the other hand, the experimental findings clearly demonstrate that the addition of coloring agents to PLA material affects not only the dimensional accuracy of the printed components, as shown above, but also their mechanical performance. Notably, across all tested speeds, the black PLA filament—which yielded superior dimensional accuracy—consistently exhibited lower tensile strength than the natural one. Interestingly, this conclusion is fully in agreement with the findings of the authors of [14,16], even though they applied moderate printing speeds (50 mm/s) and their experiments were carried out on other types of PLA filaments. The disparity in UTS between the natural and black PLA samples ranged from 0.69% at 100 mm/s to 13.16% at 400 mm/s. Also, at 300 mm/s, a substantial difference of 8.50% was observed, while differences of 4.99% and 5.46% were recorded at 200 mm/s and 500 mm/s, respectively.

With respect to the influence of printing speed on the ultimate tensile strength (UTS) of specimens produced using different filament colors, the experimental results indicated the following:

- For natural PLA, the ultimate tensile strength (UTS) exhibited a variation dependent on the printing speed, ranging from 40.90 MPa at 100 mm/s to a maximum of 46.59 MPa at 300 mm/s, corresponding to an increase of approximately 13.90%.
- In the case of black PLA, the lowest UTS was recorded at a printing speed of 400 mm/s (40.00 MPa), while the highest value was observed at 600 mm/s (43.33 MPa), reflecting a difference of 8.32%. It is worth noting that the black PLA samples printed at 300 mm/s also exhibited a relatively high ultimate tensile strength (42.94 MPa), which was close to the maximum observed value.

## 4. Conclusions

This study conducted a systematic analysis regarding the effects of elevated printing speeds, ranging from 100 mm/s to 600 mm/s, on the dimensional accuracy and ultimate tensile strength (UTS) of PLA specimens printed by FDM, utilizing two filament colors: natural and black. The key findings are as follows:

- Dimensional accuracy: The dimensional deviations in both width and thickness remained within acceptable ranges, comparable to those observed at moderate printing speeds, despite the significant increase in print rate. Among the tested speeds, 300 mm/s yielded the lowest deviations in cross-sectional area, indicating optimal dimensional fidelity for both PLA variants. Notably, black PLA exhibited superior dimensional accuracy at high speeds (≥200 mm/s) compared to natural PLA, likely due to differences in thermal conductivity and pigment composition.
- Tensile strength: The UTS followed a non-linear trend with respect to the printing speed for both materials. It improved as the speed increased from 100 mm/s up to 300 mm/s, reaching a maximum of 46.59 MPa for natural PLA and 42.94 MPa for black PLA. Beyond this point, UTS declined due to over-extrusion effects and interlayer delamination, particularly evident in the surface morphology and fracture structure analyses. Regarding the color influence, black PLA consistently showed, across all speeds, lower tensile strength than natural PLA, with differences reaching up to 13.16%. This reinforces the impact of filament pigmentation on mechanical performance, even under identical printing conditions.
- Structural insights: Microscopic analysis revealed that suboptimal speeds (too low or too high) introduced structural anomalies. Under-extrusion at lower speeds and over-extrusion at higher speeds both degraded print quality and mechanical strength. Optimal interlayer bonding and surface integrity were observed at 300 mm/s.

In conclusion, this study demonstrates that, among all the process parameters shown in Table 1, a printing speed of 300 mm/s offers the best compromise between high productivity (printing time of 9 min and 13 s compared to 26 min and 26 s at 100 mm/s printing speed), dimensional precision, and mechanical strength when using high-speed PLA filaments and 100% fan speed.

Furthermore, the use of specially developed materials in conjunction with dedicated printing systems equipped with a bilateral continuous positive airway pressure (CPAP) fan cooling mechanism may ensure dimensional accuracy and tensile strength comparable to those obtained at moderate printing speeds. These insights are crucial for practitioners aiming to scale up FDM production without compromising part quality, and they underline the necessity of considering both material composition and process parameters when optimizing high-speed FDM workflows.

## Figures and Tables

**Table 1:** FDM process parameters.

Parameters		Values
Invariable parameters	Printing head temperature, T_H_	230 °C
	Build plate temperature, T_B_	60 °C
	Layer thickness, t	0.2 mm
	Nozzle diameter, d_n_	0.40 mm
	Filament diameter, d_f_	1.75 mm
	Build orientation	YX
	Raster angle, θ	45°/−45°
	Infill density	100%
	Number of wall lines, W_L_	2
	Number of simultaneously printed samples	1 (individually printing)
	Fan speed	100%
Variable parameters	Printing speed, s_p_	100 mm/s; 200 mm/s; 300 mm/s; 400 mm/s; 500 mm/s; 600 mm/s
	Material/Filament color	ePLA Natural; ePLA Traffic Black

## Data Availability

The original contributions presented in this study are included in the article. Further inquiries can be directed to the corresponding authors.
